# Prediction of physical functioning and general health status trajectories on mortality among persons with cognitive impairment

**DOI:** 10.1186/s12877-022-03446-0

**Published:** 2022-09-21

**Authors:** Emma Zang, Xueqing Wang, Yu Shi, Bei Wu, Terri R. Fried

**Affiliations:** 1 Department of Sociology, Yale University, New Haven, CT 06520 USA; 2 Department of Biostatistics, Yale University, New Haven, CT 06520 USA; 3 Office of Population Research, Princeton University, Princeton, NJ 08540 USA; 4 School of Public and International Affairs, Princeton University, Princeton, NJ 08540 USA; 5 Rory Meyers College of Nursing, New York University, New York, NY 10010 USA; 6 Veterans Affairs Connecticut Healthcare System, West Haven, CT 06516 USA; 7 Department of Medicine, Yale School of Medicine, New Haven, CT 06520 USA

**Keywords:** Cognitive aging, Mortality, Physical functioning, Health trajectory

## Abstract

**Background:**

The concern posed by the confluence of aging and cognitive impairment is growing in importance as the U.S. population rapidly ages. As such, we sought to examine the predictive power of physical functioning (PF) and general health status (GHS) trajectories on mortality outcomes among persons with cognitive impairment (PCIs).

**Methods:**

We used group-based trajectory models to identify latent group memberships for PF trajectories in 1,641 PCIs and GHS trajectories in 2,021 PCIs from the National Health and Aging Trends Survey (2011–2018) and applied logistic regressions to predict mortality using these memberships controlling for individual characteristics.

**Results:**

We identified six trajectory groups for PF and four groups for GHS. Trajectory group memberships for both outcomes significantly predicted mortality. For PF, group memberships largely captured the average levels over time, and worse trajectories (i.e., lower baselines and faster declines) were associated with higher odds of death. The highest mortality risk was associated with the group experiencing a sharp decline early in its PF trajectory, although its average level across time was not the lowest. For GHS, we observed two groups with comparable average levels across time, but the one with a convex-shape trajectory had much higher mortality risks compared to the one with a concave-shape trajectory.

**Conclusions:**

Our findings highlighted that health trajectories predicted mortality among PCIs, not only because of general levels but also because of the shapes of declines. Close monitoring health deterioration of PCIs is crucial to understand the health burden of this population and to make subsequent actions.

**Supplementary Information:**

The online version contains supplementary material available at 10.1186/s12877-022-03446-0.

## Introduction

In the U.S. with a rapid increase of the aging population, cognitive impairment has become a major public health concern. Currently, more than 16 million people in the U.S. have various levels of cognitive impairment [1]. Studies have shown that a large portion of older adults experience cognitive decline over time [2], and have established a strong link between cognitive impairment and mortality risks [3–5] as well as between worsening cognitive trajectories and increased mortality [6–9]. Prior literature has also documented strong associations between cognitive impairment and declines in physical functioning and general health status (i.e., self-rated or proxy-rated health) [10–12]. General health status and physical functioning are some of the strongest predictors of mortality in the general population of older adults [13–15]. A handful of studies have also shown that self-rated health trajectories and physical activity trajectories significantly predict mortality among the general older adult population outside the U.S [16–18]. However, few studies have examined whether and how physical functioning and general health status, particularly trajectories, predict mortality among PCIs in the U.S. The only evidence is from studies using cross-sectional measures of general health status, which show mixed evidence on the relationship between general health status and mortality among PCIs [19, 20].

It is important to study this topic especially among PCIs because health trajectories may be more complex in this population compared to the general aging population. For example, there is substantial heterogeneity in trajectories of general health status among PCIs and declines in general health status are more common in this population compared to the general aging population in the U.S. [21, 22]. The substantially heterogeneous health trajectories may lead to substantially different mortality risks.

In addition, PCIs’ health trajectories can reveal a more complete picture of health status at different time points or at different stages of disease career that are masked by an average measure [16]. For example, some PCIs may experience a greater variability in health status over time but have the same average health status as another group of PCIs. Differences in the shape of trajectories and the rates of change are therefore not captured by an average measure of health, and these differences could potentially have an independent effect on mortality later in life.

We fill the gap in literature by exploring the relationship between trajectories of general health status and physical functioning and later-life mortality among PCIs. Using a nationally-representative dataset of U.S. Medicare beneficiaries, we apply group-based trajectory models (GBTMs) to classify PCIs into distinct trajectory latent groups and estimate their trajectories [23]. We then examine whether PCIs’ trajectory group memberships predict mortality, and whether this relationship is independent of a variety of sociodemographic and health characteristics.

## Methods

### Data and measures

This study used data from the National Health and Aging Trends Study (NHATS) from 2011 to 2018. The NHATS is a nationally representative sample of Medicare beneficiaries aged 65 and above [24, 25]. Respondents were selected using multistage sampling techniques and surveyed annually starting from 2011. Round 1 contains 8,245 home-interviewed respondents. If a sampled individual could not respond due to severe cognitive or physical impairment, a proxy respondent, either a family member or primary caregiver, was interviewed. In Round 5, the NHATS study added a refreshment sample of 4,182 respondents to maintain the representativeness. Throughout the study period, respondents were followed-up except for recorded death. On average, NHATS has response rate 89.5% for living adults and 95.5% for decreased adults [25].

Participants included in the current study were those with cognitive impairment whose cognitive scores were below the threshold for identifying Mild Cognitive Impairment (MCI) or dementia for at least two waves. Those with only one wave of data on cognitive impairment cannot provide sufficient amount of evidence for persons with MCI due to various reasons (e.g., delirium), and therefore we constructed our sample based on cognitive impairment in at least any of the two waves within the selected period. In order to be able to model their health trajectories, individuals with fewer than three rounds of follow-up after the first observation of cognitive impairment were excluded. Detailed sample construction can be found in Additional file 1: Figure S1. The final sample included 1,641 individuals for modeling physical functioning trajectories and 2,361 individuals for general health status trajectories (not every respondent had assessment-based physical functioning).

### Cognitive impairment

Cognitive impairment was defined as having either MCI or dementia. At each wave, participants were asked to perform cognitive tests to assess memory (10-item immediately and delayed recall), executive function (a clock drawing test), and orientation (questions about time and place). Following the recommended strategy by NHATS, an individual was considered to have MCI if he or she scored less or equal to 1.5 standard deviation below the mean in one of the above three domains [26]. Dementia was defined as scoring below or equal to 1.5 standard deviation below the mean in at least two domains. Dementia status was additionally determined from direct reports from respondents or based on a proxy-reported score of at least 2 on the AD8 Dementia Screening Interview [27].

### Physical functioning

Physical functioning was measured using the NHATS Expanded Short Physical Performance Battery (SPPB) [28]. Designed to capture a wide range of physical functioning, the NHATS Expanded SPPB included three components: 1) nested balance test, 2) a 3-m usual walking speed to measure locomotion, and 3) rapid chair stands to measure lower body muscle function. Each of the 3 components scores from minimum, 1, to maximum, 4, for non-missing responses. This scoring system reflects the distribution quartiles of the weighted NHATS sample [24]. A score of 0 was given if the participant was unable to stand or walk. The physical functioning measure used in this study was generated by summing up the scores of the three components, ranging from 0–12. A higher score indicates better physical functioning.

### General health status

General health status was assessed by asking respondents to self-report their general health status from 1 (*excellent*) to 5 (*poor*). 26.73% of 10,701 person-year observations for general health status were reported by a proxy respondent (the baseline proportion for proxy-report was 14.1%, as shown in Table 1). Robustness checks without proxy reports show highly consistent patterns of trajectories with the results including them. We used Diehr’s recoding strategy to transform general health status to a 15–95 scale (*excellent* = 95, *very good* = 90, *good* = 80, *fair* = 30, and *poor* = 15) to improve the interpretability of the general health status measure [29].

**Table 1:** Baseline characteristics for the general health status (GHS) and physical functioning (PF) samples

	N		Mean (SD)/ %		Missing rate	
	GHS	PF	GHS	PF	GHS	PF
Female	2021	1641	59.77%	59.72%	0.00%	0.00%
Age	2021	1641	81.05	80.76	0.00%	0.00%
			(7.59)	(7.54)		
Race/Ethnicity	1986	1616			1.73%	1.52%
White			55.99%	55.88%		
Black			30.06%	29.95%		
Other			3.98%	4.02%		
Hispanic			9.97%	10.15%		
Education attainment	1977	1608			2.18%	2.01%
< High school			43.10%	41.67%		
High school			24.73%	25.12%		
> High school			32.17%	33.21%		
# siblings	2002	1629			0.94%	0.73%
0			26.32%	26.21%		
1–3			51.80%	51.75%		
4 +			21.88%	22.04%		
# children	2021	1641			0.00%	0.00%
0			9.60%	8.78%		
1–3			53.59%	53.69%		
4 +			36.81%	37.54%		
Medicare drug coverage	1858	1520	69.32%	68.82%	8.07%	7.37%
Medicaid	1922	1568	27.63%	28.19%	4.90%	4.45%
Tricare	1961	1595	4.49%	4.95%	2.97%	2.80%
Comorbidity	1982	1611			1.93%	1.83%
0			8.17%	8.19%		
1–3			63.62%	63.63%		
4+			28.20%	28.18%		
Marital status	2017	1638			0.20%	0.18%
Never married			5.26%	4.88%		
Married/live with a partner			37.88%	38.89%		
Separated, divorced, widowed, never married			56.87%	56.23%		
Smoking regularly	1535	1272	45.60%	45.05%	24.05%	22.49%
Self-report	2021	1641	85.90%	–	0.00%	0.00%
MCI	2021	1641	38.69%	40.28%	0.00%	0.00%

Mean (SD) for continuous variables and % for categorical variables

### Covariates

We selected sociodemographic and health covariates based on prior studies on mortality risks [30–32]. They included sex, age, race/ethnicity (non-Hispanic White (hereafter “White”), non-Hispanic Black (hereafter “Black”), non-Hispanic other (hereafter “Other”), and Hispanic), educational attainment (less than high school, high school graduate, or beyond high school), number of siblings (0, 1–3, 4 +), number of children (0, 1–3, 4 +), enrollment in Medicare, enrollment in Medicare Part D, and enrollment in Tricare, comorbidity (0, 1–3, 4 +), marital status (never married, married or living with a partner, separated/divorced/widowed), smoking regularly (i.e. smoking at least 1 cigarette per day), and presence of MCI rather than dementia. Number of siblings, number of children, and comorbidity were measured as continuous variables. Using these, we constructed categorical variables to capture potential nonlinear relationships between them the mortality, based on their distributions in our sample and previous studies [33]. These covariates were time-constant and were measured at baseline.

### Statistical analyses

We applied GBTMs to identify distinct trajectory groups among PCIs and classify PCIs into these groups, assuming that there exist multiple latent subgroups with distinct trajectories within the PCI population [34]. Consistent with prior studies, we used the first observed cognitive impairment diagnosis in our data as the baseline, with years since first observed diagnosis as the time variable [21]. Based on the distributions of the general health status and physical functioning variables in our data (see Additional file 1: Figure S2), we modeled general health status using a censored normal distribution and physical functioning using a zero-inflated Poisson distribution. We first fitted models with varying numbers of latent groups and included linear, quadratic, and cubic terms to select the best functional forms of the trajectories. We selected the best fitted model based on the Bayesian information criterion (BIC) and diagnosis statistics including the average posterior probability (AvePP > 0.7) and the odds of correction classification (OCC > 5) [23]. Additional file 1: Table S1 shows the BICs and Additional file 1: Table S2 shows the diagnosis statistics. Each participant was assigned to the latent group with the highest posterior probability. All analyses were performed using the ‘traj’ package in Stata 15 [35].

After identifying latent trajectory groups and classifying PCIs into these groups, we applied multinomial logistic regression models to predict mortality in the last observed wave using latent trajectory group memberships with and without controlling for baseline covariates. To impute a small number of missing values in the covariates, we performed multiple imputations by chained equations using the Stata package ‘ice’ [36]. The regression coefficients from 10 imputed datasets were then combined based on the Rubin’s rule [37]. Results without multiple imputations, as shown in Additional file 1: Tables S3 and S4, were highly consistent. We conducted two sets of analyses. In the baseline model, we predicted mortality using group memberships without any covariates. In the second model, we additionally controlled for sociodemographic and health covariates.

## Results

### Physical functioning

**Fig. 1:**
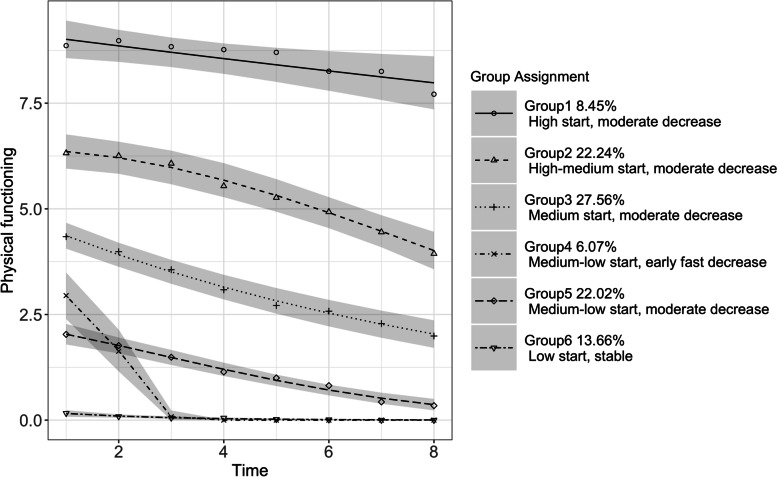
Physical functioning trajectories for individuals with cognitive impairment

We identified six latent trajectory groups, as shown in Fig. 1. Estimates of model parameters are shown in Additional file 1: Tables S5 and S6 shows the individual characteristics of each latent group. Approximately 8.45% PCIs were in Group 1 (“high start, moderate decrease”), 22.4% were in Group 2 (“high-medium start, moderate decrease”), 27.56% were in Group 3 (“medium start, moderate decrease”), 6.07% were in Group 4 (“medium–low start, early fast decrease”), 22.02% were in in Group 5 (“medium–low start, moderate decrease”), and 13.66% were in Group 6 (“low start, stable”). Groups 1 (“high start, moderate decrease”), 2 (“high-medium start, moderate decrease”), 3 (“medium start, moderate decrease”) and 5 (“medium–low start, moderate decrease”) started with baseline values 8.86, 6.32, 3.98, and 2.03, respectively, and declined at moderate rates (the slopes of the declines were -0.15, -0.34, -0.33, and -0.24, respectively). Group 4 (“medium–low start, early fast decrease”) started with a medium–low level of baseline functioning (2.95) but experienced an acute decline to an SPPB score of 0 in the first 3 years after the first observation of cognitive impairment (the slope was -1.43). Group 6 (“low start, stable”) stayed at a level of almost no physical functioning over the 8 years.

Table 2 shows the results on predicting mortality using trajectory group memberships. We used Group 1 (“high start, moderate decrease”) as the reference group because this group had the best physical functioning over time. Model 1 estimated the association without covariates. There were significant associations between group memberships and the odds of dying. The odds of dying for PCIs in Group 2 (“high-medium start, moderate decrease”) were 5.04 times as large as the odds for Group 1 (“high start, moderate decrease”). For Groups 3 (“medium start, moderate decrease”), 4 (“medium–low start, early fast decrease”), 5 (“medium–low start, moderate decrease”), 6 (“low start, stable”), the corresponding odds ratios were 8.73, 28.88, 14.84, and 21.48, respectively. This pattern persisted despite attenuated magnitudes after the inclusion of a variety of sociodemographic and health characteristics (Model 2), suggesting that the predictive power of trajectory group memberships was independent of these characteristics. In general, except for Group 4 (“medium–low start, early fast decrease”), the odds of dying increased as the group membership number increased, indicating that belonging to a trajectory group with ‘worse trajectories’ (i.e., lower baselines and/or faster declines) was associated with higher odds of dying. Group 4 (“medium–low start, early fast decrease”) was associated with the highest mortality risks, although its average level across time was higher compared to Group 6 (“low start, stable”). Among all the covariates, conditional on physical functioning trajectory group memberships, being female, Black, or Hispanic, and having MCI rather than dementia were significantly predictive of lower mortality whereas smoking regularly was significantly predictive of higher mortality.

**Table 2:** Predicting death using physical functioning trajectory group memberships, odds ratios and 95% confidence intervals

	Model 1	Model 2
Group membership (Ref. “High start, moderate decrease”)		
High-medium start, moderate decrease edecrease	5.04**	4.40**
	(1.98, 12.82)	(1.69, 11.44)
Medium start, moderate decrease	8.73***	6.59***
	(3.49, 21.87)	(2.56, 16.98)
Medium low start, early fast decrease	28.88***	19.62***
	(10.90, 76.50)	(7.14, 53.94)
Medium low start, moderate decrease	14.84***	10.81***
	(5.91, 37.25)	(4.16, 28.09)
Low start, stable	21.48***	16.03***
	(8.47, 54.50)	(6.05, 42.46)
Female		0.70*
		(0.53, 0.93)
Age		1.23
		(0.91, 1.65)
Age squared		1.00
		(1.00, 1.00)
Race/Ethnicity (Ref. White)		
Black		0.64**
		(0.47, 0.87)
Other		0.71
		(0.37, 1.37)
Hispanic		0.52**
		(0.32, 0.84)
Educational attainment (Ref. < High school)		
High school		1.02
		(0.74, 1.40)
> High school		1.21
		(0.89, 1.64)
# of siblings (Ref. 0)		
1–3		0.99
		(0.76, 1.31)
4 +		0.88
		(0.61, 1.27)
# of children (Ref. 0)		
1–3		1.02
		(0.64, 1.63)
4 +		0.87
		(0.54, 1.40)
Medicare		0.95
		(0.73, 1.23)
Medicaid		0.97
		(0.70, 1.33)
Tricare		1.35
		(0.77, 2.35)
Comorbidity (Ref. 0)		
1–3		1.09
		(0.68, 1.74)
4 +		1.34
		(0.81, 2.20)
Marital status (Ref. Never married)		
Married/live with a partner		1.28
		(0.64, 2.56)
Separated, divorced, widowed		1.14
		(0.59, 2.21)
Smoke regularly		1.33*
		(1.01, 1.75)
MCI		0.64**
		(0.50, 0.83)

^*^p < 0.05

^*^^*^p < 0.01

^*^^**^p < 0.001

*N* = 1,641

### General health status

**Fig. 2:**
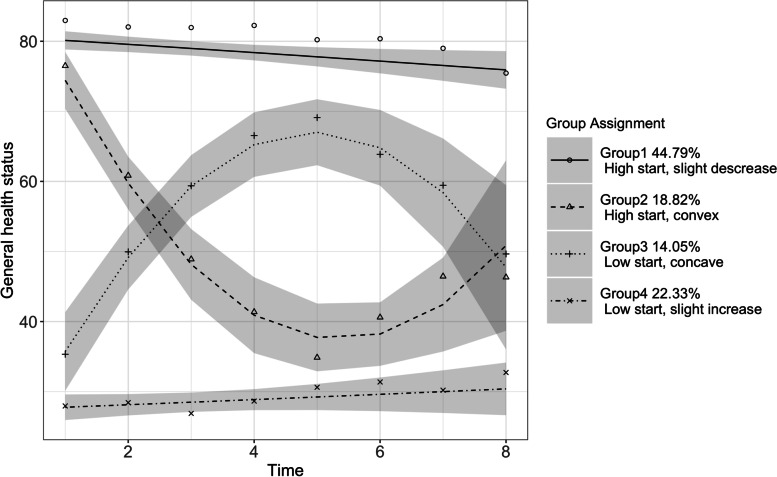
General health status trajectories for individuals with cognitive impairment

We identified four trajectory group memberships for general health status (Fig. 2). Estimates of model parameters are shown in Additional file 1: Tables S7 and S8 shows the individual characteristics of each latent group. Approximately 44.79% PCIs were in Group 1 (“high start, slight decrease”), 18.82% were in Group 2 (“high start, convex”), 14.05% were in Group 3 (“low start, concave”), 22.33% were in Group 4 (“low start, slight increase”). Group 1 (“high start, slight decrease”) started with *good* health, experienced only slight decreases over time, and still maintained *good* health at the end of our study period. Group 2 (“high start, convex”) started with close to *good* health, experienced sharp decreases first, reaching close to *fair* health, and then recovered a little after the 5th year since the first observation of cognitive impairment. Starting with a baseline value just above *fair,* Group 3 (“low start, concave”) experienced sharp increases first but then experienced declines starting from the 5th year since the first observation of cognitive impairment. At the end of the study period, this group dropped to its starting value. Group 4 (“low start, slight increase”) started with just below *fair* health and experienced increases slightly over the study period, maintaining the level of *fair* health.

Table 3 shows the results predicting mortality using general health trajectory group memberships. In Model 1 without any covariates, two of the three group memberships were significantly associated with increased odds of dying as compared to Group 1. Unlike the case for physical functioning, we did not find a general gradient in the magnitudes of the associations. Group 2 (“high start, convex”) had the greatest odds of dying, 1.79 times as large as the odds for Group 1 (“high start, slight decrease”). Group 4 (“low start, slight increase”) had a comparable magnitude, which was 1.74 times as large as the odds for Group 1 (“high start, slight decrease”). After including covariates, as shown in Model 2, the magnitudes generally became bigger for all groups, suggesting the existence of negative confounders [38]. Compared to Group 1 (“high start, slight decrease”), the odds of dying were 1.94 times, 1.46 times, and 2.32 times as large for Groups 2 (“high start, convex”), 3 (“low start, concave”) and 4 (“low start, slight increase”), respectively. The average level of general health status for Groups 2 (“high start, convex”) and 3 (“low start, concave”) were comparable with values of 53.5 and 54.3. Conditional on general health status trajectory group memberships, being Blacks or Hispanics, having at least 4 children, self-report, and having MCI rather than dementia were significantly predictive of lower mortality, whereas smoking regularly was significantly predictive of higher mortality. These results suggest that the relationship between these covariates and mortality was not completely mediated by general health status trajectories.

**Table 3:** Predicting death using general health status trajectory group memberships, odds ratios and 95% confidence intervals

	Model 1	Model 2
Group membership (Ref. high start, slight decrease)		
High start, convex	1.79***	1.94***
	(1.40, 2.30)	(1.47, 2.55)
Low start, concave	1.19	1.46*
	(0.88, 1.61)	(1.04, 2.02)
Low start, slight increase	1.74***	2.32***
	(1.37, 2.20)	(1.74, 3.09)
Female		0.93
		(0.73, 1.18)
Age		1.27
		(0.98, 1.65)
Age squared		1.00
		(1.00, 1.00)
Race/Ethnicity (Ref. White)		
Black		0.68**
		(0.54, 0.91)
Other		0.63
		(0.35, 1.11)
Hispanic		0.50**
		(0.33, 0.76)
Educational attainment (Ref. < High school)		
High school		1.05
		(0.79, 1.39)
> High school		1.26
		(0.97, 1.65)
# of siblings (Ref. 0)		
1–3		0.95
		(0.75, 1.22)
4 +		0.84
		(0.61, 1.16)
# of children (Ref. 0)		
1–3		0.74
		(0.51, 1.07)
4 +		0.67*
		(0.46, 1.00)
Medicare		0.96
		(0.76, 1.21)
Medicaid		0.93
		(0.70, 1.22)
Tricare		1.09
		(0.65, 1.83)
Comorbidity (Ref. 0)		
1–3		1.24
		(0.83, 1.85)
4 +		1.39
		(0.90, 2.14)
Marital status (Ref. Never married)		
Married/live with a partner		1.10
		(0.63, 1.90)
Separated, divorced, widowed		0.99
		(0.58, 1.68)
Smoke regularly		1.52**
		(1.17, 1.97)
Self-report		0.61**
		(0.46, 0.82)
MCI		0.59***
		(0.47, 0.73)

^*^p < 0.05; ** *p* < 0.01; *** *p* < 0.001. *N* = 2,021

## Discussion

This study examined whether and how trajectory group memberships for PCIs’ physical functioning and general health status predict mortality risks. Using group-based trajectory modeling, we identified six distinct trajectory groups for physical functioning and four groups for general health status among PCIs. We found that trajectory group memberships for both physical functioning and general health status significantly predicted mortality. For physical functioning, except for Group 4 (“medium–low start, early fast decrease”), group memberships were largely reflective of the average levels across time among PCIs, and worse trajectories (i.e., lower baselines and faster declines) were associated with higher odds of death. However, although Group 4 (“medium–low start, early fast decrease”) had a higher average level across time than Group 6 (“low start, stable”), the former had much higher mortality. For general health status, we observed two groups with comparable average levels across time, but the one with convex-shape trajectories had higher mortality risks compared to the one with concave-shape trajectories. For both health outcomes, the patterns largely persisted after the inclusion of various sociodemographic and health characteristics.

Our findings on physical functioning highlight the importance of considering trajectories, capturing information on both the general level of physical functioning and the rate of change over time. The results provide examples in which the shape of health deterioration is more predictive of mortality than are the average levels across time. For example, Group 4 (“medium low start, early fast decrease”) had higher mortality risks than Group 6 (“low start, stable”), which had lower average levels across time. Group 4 also had substantially higher mortality risks than Group 5 (“medium low start, moderate decrease”). There are several explanations to consider. One is that the declining function of Group 4 (“medium low start, early fast decrease”) is a consequence of worsening chronic conditions, which is responsible for increased mortality risks. Alternatively, it is possible that there is a negative feedback loop for some individuals. In this case, declining functioning decreases resilience, causing worsening of chronic conditions, which in turn further decreasing functioning. In any case, these findings suggest that rapidly declining functioning is robust indicator of increased mortality risks. The clinical challenge requiring additional research is understanding when interventions can change this trajectory and when these trajectories are irreversible and should trigger consideration of a more palliative approach to care.

Results for general health status similarly highlight the importance of examining trajectories. For example, despite comparable average levels across time, Group 2 (“high start, convex”) had higher mortality risks than Group 3 (“low start, concave”). The convex [21, 39] and concave [21, 40, 41] shapes of general health status trajectories have also been found in previous studies examining populations not specific to PCIs. For example, a study reported two distinctly-shaped convex trajectories of self-rated health [41]. Different from the consistently declining patterns we observed for physical functioning, the convex and concave shapes of trajectories we observed for general health status may have reflected the fact that general health status was mostly self-rated (only a small percent was reported by proxy). Self-rated health is a more inclusive measurement of one’s health status compared to objective measures [42]. An individual’s self-report encompasses a range of factors, including but not limited to one’s history of health, levels of social support, and psychological factors [42]. These factors included in the measurement of self-rated health could potentially explain why the trajectories of general health status change non-linearly. In particular, older adults tend to put more weights on psychological factors when self-accessing health conditions [43]. In general, the relationship between respondent- or proxy-reported general health status and mortality is more complex than the one for assessment-based physical functioning. Future research is needed to determine the mechanism by which different trajectories are associated with differential risks.

We also found that being female, Black, or Hispanic, and having MCI rather than dementia were significantly predictive of lower mortality whereas smoking regularly was significantly predictive of higher mortality, conditional on physical functioning trajectories. Conditional on general health status trajectories, being Blacks or Hispanics, having at least 4 children, self-report, having MCI rather than dementia, and not smoking regularly were significantly predictive of lower mortality. Other covariates were not significant likely because trajectories largely mediated the relationship between them and mortality. One explanation for the lower mortality risks among Blacks and Hispanics is mortality selection: Blacks and Hispanics disproportionately die early before entering our sample, and therefore the ones left in our sample are positively selected in terms of health. The finding that persons with MCI had lower mortality risks than those with dementia, conditional on trajectory group memberships, adds to the literature demonstrating associations between declining cognition and increased mortality [44, 45].

Our findings may add to the literature in several ways. First, we found that physical functioning trajectory group memberships were highly predictive of mortality, especially when we compared the magnitudes of the coefficients to those for general health status. Indeed, the predictive power was so strong that physical functioning trajectories might have overpowered the many other known risk factors in the models. The comparison between Groups 4 (“medium–low start, early fast decrease”) and 6 (“low start, stable”) further confirms the notion that the shape of trajectories matters to mortality risks on top of the average levels across time. Therefore, clinicians should pay particular attention to the mortality risks of those who experienced sharp declines in physical functioning in a relatively short period of time.

Second, prior literature has shown that general health status is associated with mortality in the general population [42, 46] but such association was not found among PCIs [47]. Nielsen et al. [47] raised an explanation that general health status is not a valid health indicator among PCIs due to “loss of insight”. However, alternatively, the lack of predictive power of general health status to mortality may be driven by the lack of information on changes of general health status over time, and no previous studies have explored whether and how its trajectories predict mortality among PCIs. Our results suggest that trajectories of general health status are a robust predictor of mortality among PCIs because they capture both the average health level across time and the shape of health deterioration. Therefore, our findings underscore the need to collect general health status trajectory data, rather than cross-sectional ones, for policymakers to better estimate mortality risks for the PCI population.

Third, our results on general health status without adjusting for covariates also suggest that those who perceived their health to be declining over time were at about the same risk for mortality as those with persistently poor general health status, while those who perceived their health to be improving were not statistically different from those who had persistently high general health status. Therefore, previous studies that only measured general health status at the baseline when predicting mortality can lead to misleading results. For example, the baseline value for Group 2 (“high start, convex”) was much higher than that for Group 3 (“high start, concave”), but the former had higher mortality risks than the latter due to differential shapes in the trajectories. When comparing these two groups (see Additional file 1: Table S8), the former tended to be White and have relatively higher socioeconomic status and fewer comorbidities compared to the latter. Future studies are needed to examine what determines the differential shapes of trajectories between these two groups. Perhaps mental health is a determinant here, as Black Americans tended to be happier than Whites [48].

Our study has several limitations. First, although we controlled for a variety of individual sociodemographic and health characteristics when predicting mortality, we may still have missed some important characteristics, such as living arrangements. Second, our sample restriction criteria may lead to unhealthy individuals less likely being included in our sample. Individuals excluded because of having fewer than three rounds of data were more likely to have low socioeconomic statuses (see Additional file 1: Table S9). Lastly, we examined the predictive power of general health status and physical functioning trajectories separately. By doing so, our findings are straightforward to interpret and can be compared with findings from previous studies which tend to focus on a single mortality predictor. In addition, these two trajectories may play different roles in predicting mortality, such as the fact that physical functioning trajectories were particularly predictive of mortality among PCIs. However, considering health is a multidimensional construct, future studies may consider modeling joint trajectories of multiple health dimensions (e.g., general health status, physical functioning, depressive symptoms) to predict mortality among PCIs.

Despite these limitations, this study suggests several avenues for future research and policy making. Our findings highlighted that health trajectories predicted mortality among PCIs, because of both the general levels and the shapes of declines. Close monitoring health deterioration of PCIs is crucial to understand the health burden of this population and to make subsequent actions. From a clinical perspective, the strong association between a rapid functional decline and mortality suggests that a change in function should become a routine measurement in the care of PCIs. A rapid change can then serve as a strong prognostic marker, either signaling the need for more intensive interventions, if the goal is to try to extend life, or for a switch to palliative care, if the goal is something other than life extension. Future research is also needed to have a better understanding of the components that go into self-rated general health, in order to identify modifiable factors that may be protective of mortality.

## Supplementary Information

**Additional file 1: Figure S1.** Flow chart of sample restrictions. **Figure S2.** Baseline distributions of physical functioning and general health status. **Table S1.** Model selection of assessment-based physical functioning and general health status trajectories models. **Table S2.** Assessment-based physical functioning and general health status model diagnosis. **Table S3.** Risk factors of physical functioning trajectories for physical functioning (Odds Ratios and 95% Confidence Interval), without multiple imputation. **Table S4.** Risk factors of general health status trajectories (Odds Ratios and 95% Confidence Interval), without multiple imputation. **Table S5.** Group-based assessment-based trajectory model of physical functioning. **Table S6.** Baseline sample characteristics by trajectory group of physical functioning. **Table S7.** Group-based assessment-based trajectory model of general health status. **Table S8.** Baseline sample characteristics by trajectory group of general health status. **Table S9.** Comparison of the individuals included in the sample with individuals excluded from the sample because of having fewer than three rounds of observations.

## Data Availability

Part of the datasets generated and analyzed during the current study are not publicly available because they are restricted access (see NHATS website: https://nhats.org/researcher/nsoc), but are available from the corresponding author on reasonable request and with NHATS’ permission.

## References

[1] Cogntive Impairement: Call for Action, Now! [https://www.cdc.gov/aging/pdf/cognitive_impairment/cogimp_poilicy_final.pdf]

[2] Park HL, O'Connell JE, Thomson RG (2003). A systematic review of cognitive decline in the general elderly population. Int J Geriatr Psychiatry.

[3] van de Vorst IE, Koek HL, Stein CE, Bots ML, Vaartjes I (2016). Socioeconomic disparities and mortality after a diagnosis of dementia: results from a nationwide registry linkage study. Am J Epidemiol.

[4] Zilkens RR, Davis WA, Spilsbury K, Semmens JB, Bruce DG (2013). Earlier age of dementia onset and shorter survival times in dementia patients with diabetes. Am J Epidemiol.

[5] Helmer C, Joly P, Letenneur L, Commenges D, Dartigues J-F (2001). Mortality with dementia: results from a french prospective community-based Cohort. Am J Epidemiol.

[6] Taniguchi Y, Kitamura A, Ishizaki T, Fujiwara Y, Shinozaki T, Seino S, Mitsutake S, Suzuki H, Yokoyama Y, Abe T (2019). Association of trajectories of cognitive function with cause-specific mortality and medical and long-term care costs. Geriatr Gerontol Int.

[7] Yaffe K, Peltz CB, Ewing SK, McCulloch CE, Cummings SR, Cauley JA, Hillier TA, Ensrud KE (2016). Long-term cognitive trajectories and mortality in older women. J Gerontol: Series A.

[8] Chen NW, Mutambudzi M, Markides KS (2022). Trajectories of concurrent depressive symptoms and cognitive function on health outcomes and mortality among older Mexican Americans. Arch Gerontol Geriatr.

[9] Hu X, Gu S, Sun X, Gu Y, Zhen X, Li Y, Huang M, Wei J, Dong H (2019). Cognitive ageing trajectories and mortality of Chinese oldest-old. Arch Gerontol Geriatr.

[10] Clouston SAP, Brewster P, Kuh D, Richards M, Cooper R, Hardy R, Rubin MS, Hofer SM (2013). The dynamic relationship between physical function and cognition in longitudinal aging cohorts. Epidemiol Rev.

[11] Auyeung TW, Kwok T, Lee J, Leung PC, Leung J, Woo J (2008). Functional decline in cognitive impairment— the relationship between physical and cognitive function. Neuroepidemiology.

[12] Stephan Y, Sutin AR, Luchetti M, Aschwanden D, Terracciano A (2021). Self-rated health and incident dementia over two decades: Replication across two cohorts. J Psychiatr Res.

[13] Wu C, Geldhof GJ, Xue Q-L, Kim DH, Newman AB, Odden MC (2018). Development, construct validity, and predictive validity of a continuous frailty scale: results from 2 large US Cohorts. Am J Epidemiol.

[14] Lima-Costa MF, Cesar CC, Chor D, Proietti FA (2011). Self-rated health compared with objectively measured health status as a tool for mortality risk screening in older adults: 10-year follow-up of the Bambuí Cohort study of aging. Am J Epidemiol.

[15] Smits CHM, Deeg DJH, Kriegsman DMW, Schmand B (1999). Cognitive functioning and health as determinants of mortality in an older population. Am J Epidemiol.

[16] Zimmer Z, Martin LG, Jones BL, Nagin DS (2014). Examining late-life functional limitation trajectories and their associations with underlying onset, recovery, and mortality. J Gerontol B Psychol Sci Soc Sci.

[17] Mok A, Khaw K-T, Luben R, Wareham N, Brage S (2019). Physical activity trajectories and mortality: population based cohort study. BMJ.

[18] Falconer J, Quesnel-Vallée A (2017). Pathway from poor self-rated health to mortality: explanatory power of disease diagnosis. Soc Sci Med.

[19] Walker JD, Maxwell CJ, Hogan DB, Ebly EM (2004). Does self-rated health predict survival in older persons with cognitive impairment?. J Am Geriatr Soc.

[20] Phung TKT, Siersma V, Vogel A, Waldorff FB, Waldemar G (2018). Self-rated versus caregiver-rated health for patients with mild dementia as predictors of patient mortality. Am J Geriatr Psychiatry.

[21] Zang E, Guo A, Pao C, Lu N, Wu B, Fried TR (2022). Trajectories of general health status and depressive symptoms among persons with cognitive impairment in the United States. J Aging Health.

[22] Feenstra M, van Munster BC, Vroomen JLM, de Rooij SE, Smidt N (2020). Trajectories of self-rated health in an older general population and their determinants: the lifelines cohort study. BMJ Open.

[23] Nagin DS (2005). Group-based modeling of development.

[24] National Health and Aging Trends Study User Guide: Rounds 1–9 Beta Release [https://nhats.org/sites/default/files/2021-04/NHATS_User_Guide_R9_Final_Release_0.pdf]

[25] Freedman VA, Kasper JD (2019). Cohort Profile: The National Health and Aging Trends Study (NHATS). Int J Epidemiol.

[26] Kasper JD, Freedman VA (2021). National health and aging trends study user guide: rounds 1–9 final release.

[27] Galvin JE, Roe CM, Coats MA, Morris JC (2007). Patient's Rating of Cognitive Ability: Using the AD8, a Brief Informant Interview, as a Self-rating Tool to Detect Dementia. Arch Neurol.

[28] Guralnik JM, Simonsick EM, Ferrucci L, Glynn RJ, Berkman LF, Blazer DG, Scherr PA, Wallace RB (1994). A short physical performance battery assessing lower extremity function: association with self-reported disability and prediction of mortality and nursing home admission. J Gerontol.

[29] Diehr P, Patrick DL, Spertus J, Kiefe CI, McDonell M, Fihn SD (2001). Transforming self-rated health and the SF-36 scales to include death and improve interpretability. Med Care.

[30] Stolz E, Hoogendijk EO, Mayerl H, Freidl W (2020). Frailty changes predict mortality in 4 longitudinal studies of aging. J Gerontol: Series A.

[31] Bassuk SS, Berkman LF, Amick BC (2002). Socioeconomic status and mortality among the elderly: findings from four US communities. Am J Epidemiol.

[32] Fried LP, Kronmal RA, Newman AB, Bild DE, Mittelmark MB, Polak JF, Robbins JA, Gardin JM (1998). Group ftCHSCR: risk factors for 5-year mortality in older adults: the cardiovascular health Study. JAMA.

[33] Zang E, Guo A, Pao C, Lu N, Wu B, Fried TR (2022). Trajectories of general health status and depressive symptoms among persons with cognitive impairment in the United States. J Aging Health.

[34] Nagin DS, Odgers CL (2010). Group-based trajectory modeling in clinical research. Annu Rev Clin Psychol.

[35] Jones BL, Nagin DS (2013). A note on a stata plugin for estimating group-based trajectory models. Sociol Methods Res.

[36] Royston P, White IR. Multiple Imputation by Chained Equations (MICE): implementation in Stata. J Stat Soft. 2011;45(4):1–20.

[37] Rubin DB (1987). Multiple imputation for nonresponse in surveys.

[38] Mehio-Sibai A, Feinleib M, Sibai TA, Armenian HK (2005). A positive or a negative confounding variable? A simple teaching aid for clinicians and students. Ann Epidemiol.

[39] Ayyagari P, Ullrich F, Malmstrom TK, Andresen EM, Schootman M, Miller JP, Miller DK, Wolinsky FD (2012). Self-rated health trajectories in the African American health cohort. PLoS ONE.

[40] Stenholm S, Virtanen M, Pentti J, Oksanen T, Kivimäki M, Vahtera J (2020). Trajectories of self-rated health before and after retirement: evidence from two cohort studies. Occup Environ Med.

[41] Miller TR, Wolinsky FD (2007). Self-rated health trajectories and mortality among older adults. J Gerontol B Psychol Sci Soc Sci.

[42] Idler EL, Benyamini Y (1997). Self-rated health and mortality: a review of twenty-seven community studies. J Health Soc Behav.

[43] Mossey JM, Shapiro E (1982). Self-rated health: a predictor of mortality among the elderly. Am J Public Health.

[44] Lv X, Li W, Ma Y, Chen H, Zeng Y, Yu X, Hofman A, Wang H (2019). Cognitive decline and mortality among community-dwelling Chinese older people. BMC Med.

[45] Hui J, Wilson R, Bennett D, Bienias J, Gilley D, Evans D (2003). Rate of cognitive decline and mortality in Alzheimer’s disease. Neurology.

[46] DeSalvo KB, Bloser N, Reynolds K, He J, Muntner P (2006). Mortality prediction with a single general self-rated health question. J Gen Intern Med.

[47] Nielsen ABS, Siersma V, Waldemar G, Waldorff FB (2016). Poor self-rated health did not increase risk of permanent nursing placement or mortality in people with mild Alzheimer’s disease. BMC Geriatr.

[48] Tang F, Jang H, Rauktis MB, Musa D, Beach S (2019). The race paradox in subjective wellbeing among older Americans. Ageing Soc.

