# Supplementary material for: Prediction of physical functioning and general health status trajectories on mortality among persons with cognitive impairment
Source: BMC Geriatr. 2022 Sep 21;22:766. doi: 10.1186/s12877-022-03446-0 (PMC9494770; doi:10.1186/s12877-022-03446-0)
Supplement: Supplementary file 1 — Additional file 1: Figure S1. Flow chart of sample restrictions. Figure S2. Baseline distributions of physical functioning and general health status. Table S1. Model selection of assessment-based physical functioning and general health status trajectories models. Table S2. Assessment-based physical functioning and general health status model diagnosis. Table S3. Risk factors of physical functioning trajectories for physical functioning (Odds Ratios and 95% Confidence Interval), without multiple imputation. Table S4. Risk factors of general health status trajectories (Odds Ratios and 95% Confidence Interval), without multiple imputation. Table S5. Group-based assessment-based trajectory model of physical functioning. Table S6. Baseline sample characteristics by trajectory group of physical functioning. Table S7. Group-based assessment-based trajectory model of general health status. Table S8. Baseline sample characteristics by trajectory group of general health status. Table S9. Comparison of the individuals included in the sample with individuals excluded from the sample because of having fewer than three rounds of observations. [file 12877_2022_3446_MOESM1_ESM.docx]

**Appendix Figures**

Figure S1. Flow chart of sample restrictions

12,427 participants

2,214 with less than two rounds of cognitive impairment

4,892 participants

7,535 without diagnosis of cognitive impairment

Exclude individuals with less than three-round data for assessment-based physical functioning (737 obs) and individuals with less than three-round data for general health status (357 obs)

2,378 participants

1,641 participants with physical functioning and 2,021 participants with general health status

300 with less than three rounds of data

2,678 participants

Figure S2. Baseline distributions of physical functioning and general health status

**
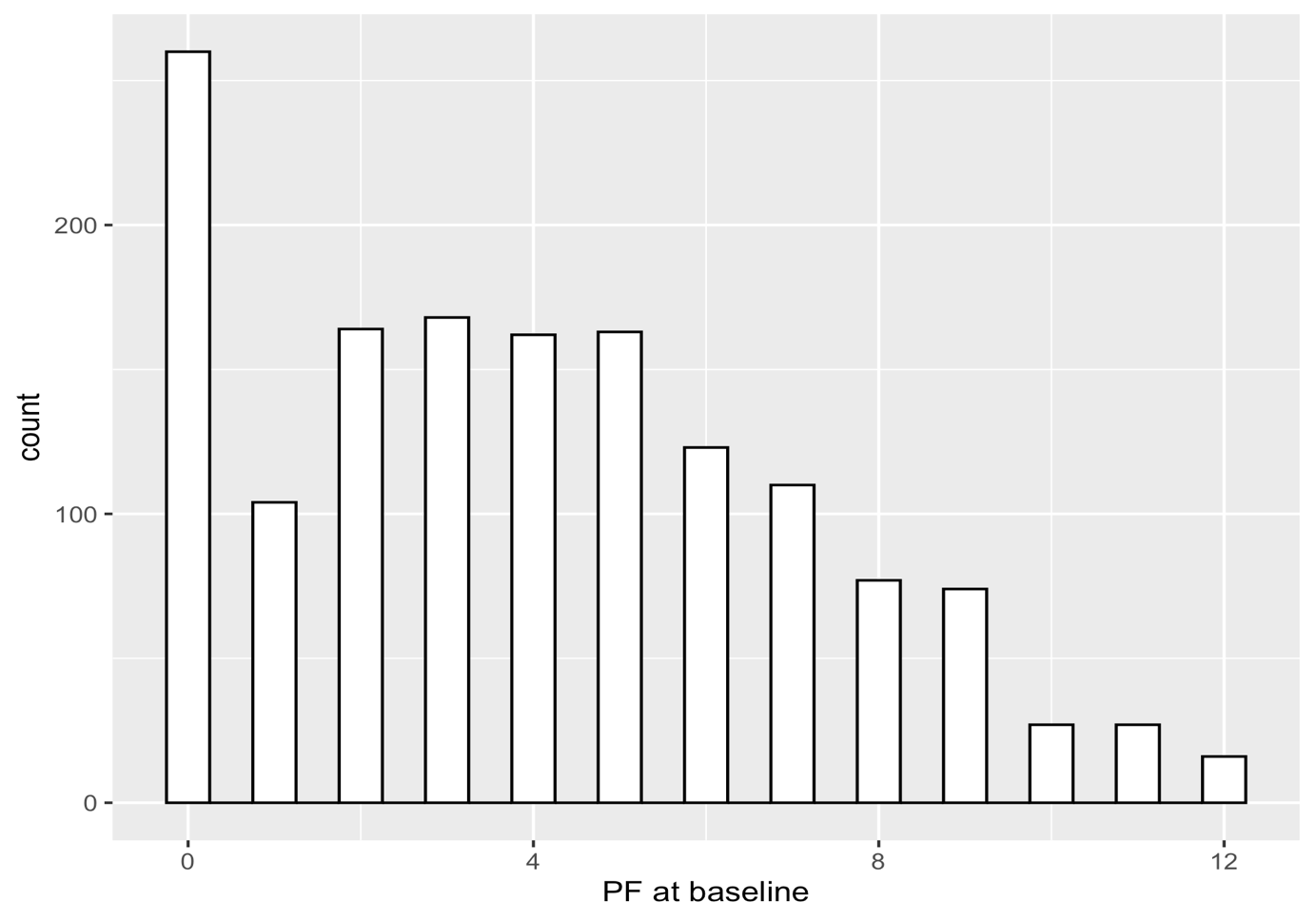
**

**
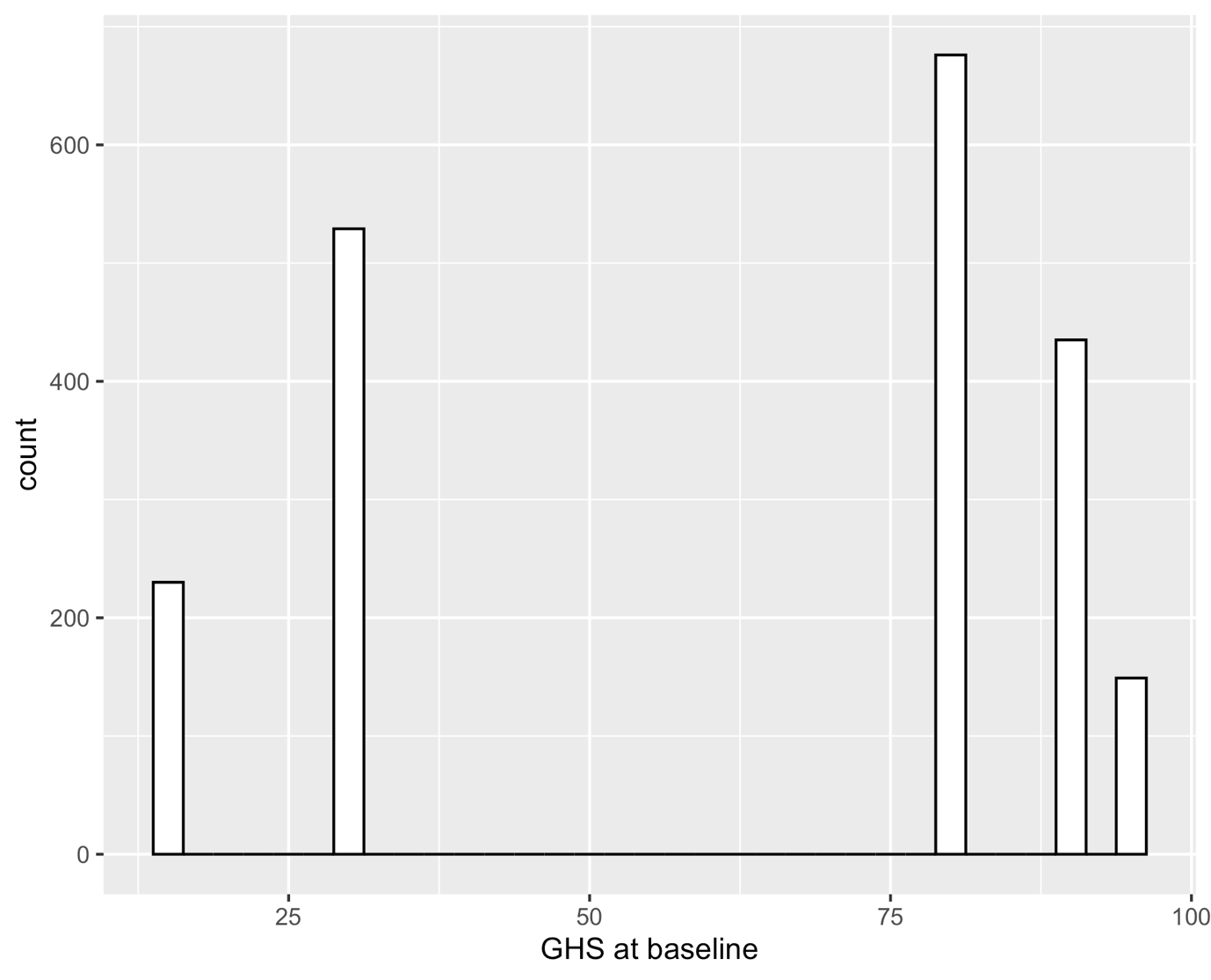
**

**Appendix Tables**

Table S1. Model selection of assessment-based physical functioning and general health status trajectories models

| Health outcomes | | Functional form | | | | | | *Log likelihood* | *BIC* |
| --- | --- | --- | --- | --- | --- | --- | --- | --- | --- |
| PH | Group 1 | | Group 2 | Group 3 | Group 4 | Group 5 | Group 6 |  |  |
|  | 1 | | 1 |  |  |  |  | -15894.38 | -15912.89 |
|  | 1 | | 1 | 2 |  |  |  | -14957.00 | -14990.32 |
|  | 1 | | 1 | 1 | 1 |  |  | -14635.20 | -14675.92 |
|  | 1 | | 1 | 1 | 2 | 1 |  | -14547.16 | -14614.05 |
|  | 1 | | 2 | 2 | 1 | 2 | 1 | -14397.85 | -14488.50 |
| GHS | 1 | | 1 |  |  |  |  | -40678.69 | -40651.10 |
|  | 1 | | 1 | 1 |  |  |  | -40480.94 | -40522.32 |
|  | 1 | | 2 | 2 | 1 |  |  | -40329.25 | -40393.61 |
|  | 1 | | 2 | 2 | 1 | 1 |  | -40310.22 | -40388.38 |
|  | 1 | | 2 | 2 | 2 | 1 | 1 | -40237.26 | -40333.81 |

Source: National Health and Aging Trends Study (NHATS), 2011-2018

Notes: BIC = log(L)-0.5*log(n)*k. The numbers 1, 2 and 3 for a certain group indicate whether the functional form of the health trajectory is linear, quadratic or cubic; All combinations of linear, quadratic, and cubic functional forms were tested. Due to page limits, we only report the best fitted model for each number of groups here. A BIC closer to 0 or a larger log likelihood indicates a better fit. For GHS, although 5 and 6 groups have better BICs, we chose 4 groups as the best model because some groups were not well-populated (i.e., with below 2% individuals) when we had 5 or 6 groups.

Table S2. Assessment-based physical functioning and general health status model diagnosis

| Group | Mean posterior probabilities (AvePP) | Median posterior probabilities | Actual group size (%) | Predicted group size (%) | Odds of correct classification (OCC) |
| --- | --- | --- | --- | --- | --- |
| PH |  |  |  |  |  |
| 1 | 0.86 | 0.93 | 8.39% | 7.27% | 68.81 |
| 2 | 0.78 | 0.79 | 22.22% | 22.30% | 12.21 |
| 3 | 0.78 | 0.80 | 27.66% | 28.21% | 9.28 |
| 4 | 0.68 | 0.68 | 6.03% | 6.34% | 32.66 |
| 5 | 0.82 | 0.88 | 22.01% | 21.51% | 16.64 |
| 6 | 0.91 | 0.98 | 13.68% | 14.26% | 65.46 |
| GHS |  |  |  |  |  |
| 1 | 0.92 | 0.99 | 44.79% | 45.37% | 14.19 |
| 2 | 0.77 | 0.79 | 18.82% | 19.45% | 14.14 |
| 3 | 0.77 | 0.80 | 14.05% | 12.96% | 20.73 |
| 4 | 0.90 | 0.96 | 22.33% | 22.22% | 31.99 |

Notes: OCC = [AvePP/(1 - AvePP)]/[$\hat{\pi}/(1-\hat{\pi})$], $\hat{\pi}$ is the estimated posterior probability. An AvePP greater than 0.7 and an OCC greater than 5 are indicative of good model fit. Median posterior probabilities are an alternative statistic to AvePP. Actual group size and predicted group size indicate the observed and precited percentages of individuals in the sample belonging to each latent group. Following previous studies, each latent group should have at least 2% of individuals.

Table S3. Risk factors of physical functioning trajectories for physical functioning (Odds Ratios and 95% Confidence Interval), without multiple imputation

|  | Model 1 | Model 2 |
| --- | --- | --- |
| Group membership (Ref. Group 1) |  |  |
| Group 2 | 5.04** | 4.20** |
|  | (1.98, 12.82) | (1.59, 11.14) |
| Group 3 | 8.73*** | 6.49*** |
|  | (3.49, 21.87) | (2.46, 17.13) |
| Group 4 | 28.88*** | 25.11*** |
|  | (10.90, 76.50) | (8.52, 74.00) |
| Group 5 | 14.84*** | 11.29*** |
|  | (5.91, 37.25) | (4.21, 30.22) |
| Group 6 | 21.48*** | 17.85*** |
|  | (8.47, 54.50) | (6.49, 49.10) |
| Female |  | 0.57** |
|  |  | (0.40, 0.79) |
| Age |  | 1.09 |
|  |  | (0.75, 1.58) |
| Age squared |  | 1.00 |
|  |  | (1.00, 1.00) |
| Race/Ethnicity (Ref. White) |  |  |
| Black |  | 0.68* |
|  |  | (0.48, 0.96) |
| Other |  | 0.73 |
|  |  | (0.33, 1.61) |
| Hispanic |  | 0.65 |
|  |  | (0.38, 1.10) |
| Educational attainment (Ref. < High school) | | |
| High school |  | 1.25 |
|  |  | (0.87, 1.80) |
| > High school |  | 1.33 |
|  |  | (0.93, 1.89) |
| # of siblings (Ref. 0) |  |  |
| 1-3 |  | 1.07 |
|  |  | (0.77, 1.48) |
| 4+ |  | 0.75 |
|  |  | (0.49, 1.15) |
| # of children (Ref. 0) |  |  |
| 1-3 |  | 1.33 |
|  |  | (0.77, 2.31) |
| 4+ |  | 1.07 |
|  |  | (0.61, 1.89) |
| Medicare |  | 1.04 |
|  |  | (0.78, 1.40) |
| Medicaid |  | 0.96 |
|  |  | (0.67, 1.37) |
| Tricare |  | 1.59 |
|  |  | (0.84, 3.03) |
| Comorbidity (Ref. 0) |  |  |
| 1-3 |  | 1.04 |
|  |  | (0.61, 1.77) |
| 4+ |  | 1.44 |
|  |  | (0.81, 2.55) |
| Marital status (Ref. Never married) | | |
| Married/live with a partner |  | 1.01 |
|  |  | (0.44, 2.31) |
| Separated, divorced, widowed |  | 0.94 |
|  |  | (0.42, 2.10) |
| Smoke regularly |  | 1.36* |
|  |  | (1.01, 1.82) |
| MCI |  | 0.59*** |
|  |  | (0.44, 0.79) |

Note: * p<0.05; ** p<0.01; *** p<0.001. N=1,164. Odds Ratios were reported.

Table S4. Risk factors of general health status trajectories (Odds Ratios and 95% Confidence Interval), without multiple imputation

|  | Model 1 | Model 2 |
| --- | --- | --- |
| Group membership (Ref. Group 1) |  |  |
| Group 2 | 1.79*** | 2.39*** |
|  | (1.40, 2.30) | (1.71, 3.34) |
| Group 3 | 1.19 | 1.66* |
|  | (0.88, 1.61) | (1.13, 2.44) |
| Group 4 | 1.74*** | 2.92*** |
|  | (1.37, 2.20) | (2.06, 4.15) |
| Female |  | 0.84 |
|  |  | (0.63, 1.13) |
| Age |  | 1.10 |
|  |  | (0.79, 1.53) |
| Age squared |  | 1.00 |
|  |  | (1.00, 1.00) |
| Race/Ethnicity (Ref. White) |  |  |
| Black |  | 0.73* |
|  |  | (0.53, 0.99) |
| Other |  | 0.51 |
|  |  | (0.25, 1.05) |
| Hispanic |  | 0.56* |
|  |  | (0.35, 0.91) |
| Educational attainment (Ref. < High school) | | |
| High school |  | 1.19 |
|  |  | (0.86, 1.66) |
| > High school |  | 1.30 |
|  |  | (0.95, 1.79) |
| # of siblings (Ref. 0) |  |  |
| 1-3 |  | 1.06 |
|  |  | (0.79, 1.41) |
| 4+ |  | 0.72 |
|  |  | (0.49, 1.05) |
| # of children (Ref. 0) |  |  |
| 1-3 |  | 0.95 |
|  |  | (0.61, 1.49) |
| 4+ |  | 0.81 |
|  |  | (0.50, 1.29) |
| Medicare |  | 0.99 |
|  |  | (0.76, 1.29) |
| Medicaid |  | 1.00 |
|  |  | (0.72, 1.38) |
| Tricare |  | 1.26 |
|  |  | (0.69, 2.30) |
| Comorbidity (Ref. 0) |  |  |
| 1-3 |  | 1.26 |
|  |  | (0.78, 2.03) |
| 4+ |  | 1.58 |
|  |  | (0.94, 2.65) |
| Marital status (Ref. Never married) | | |
| Married/live with a partner |  | 1.02 |
|  |  | (0.53, 1.97) |
| Separated, divorced, widowed |  | 0.87 |
|  |  | (0.46, 1.66) |
| Smoke regularly |  | 1.54** |
|  |  | (1.19, 1.99) |
| Self-report |  | 0.52** |
|  |  | (0.36, 0.75) |
| MCI |  | 0.57*** |
|  |  | (0.44, 0.75) |

Note: * p<0.05; ** p<0.01; *** p<0.001. N = 1,393. Reference group is Group 1 (High start, slight decrease.

Table S5. Group-based assessment-based trajectory model of physical functioning

| Group | Parameter | Estimate | Standard Error | T for H0:  Parameter=0 | Prob>\|T\| |
| --- | --- | --- | --- | --- | --- |
| 1 | Intercept | 2.24 | 0.03 | 75.39 | 0.00 |
|  | Linear | -0.02 | 0.01 | -2.47 | 0.01 |
| 2 | Intercept | 1.89 | 0.04 | 43.33 | 0.00 |
|  | Linear | -0.00 | 0.02 | -0.10 | 0.92 |
|  | Quadratic | -0.01 | 0.00 | -2.41 | 0.02 |
| 3 | Intercept | 1.61 | 0.04 | 42.74 | 0.00 |
|  | Linear | -0.11 | 0.01 | -10.23 | 0.00 |
| 4 | Intercept | -0.76 | 1.00 | -0.76 | 0.45 |
|  | Linear | 3.08 | 1.48 | 2.08 | 0.04 |
|  | Quadratic | -1.22 | 0.48 | -2.53 | 0.01 |
| 5 | Intercept | 0.84 | 0.10 | 8.74 | 0.00 |
|  | Linear | -0.09 | 0.06 | -1.44 | 0.15 |
|  | Quadratic | -0.02 | 0.01 | -2.06 | 0.04 |
| 6 | Intercept | -1.32 | 0.35 | -3.78 | 0.00 |
|  | Linear | -0.50 | 0.14 | -3.54 | 0.00 |

Table S6. Baseline sample characteristics by trajectory group of physical functioning

|  | High start, moderate decrease | High-medium start, moderate decrease | Medium start, moderate decrease | Medium-low start, early fast decrease | Medium-low start, moderate decrease | Low start, stable |
| --- | --- | --- | --- | --- | --- | --- |
| Female | 36.07% | 47.96% | 59.35% | 59.05% | 69.69% | 76.50% |
| Age | 73.98 (5.75) | 78.41 (6.82) | 80.86 (6.66) | 82.68 (8.11) | 83.04 (7.39) | 83.48 (7.80) |
| Race/Ethnicity |  |  |  |  |  |  |
| White | 63.93% | 56.16% | 58.15% | 58.82% | 56.07% | 44.93% |
| Black | 20.49% | 28.49% | 28.41% | 26.47% | 29.77% | 42.29% |
| Other | 9.84% | 3.56% | 3.08% | 3.92% | 3.75% | 3.96% |
| Hispanic | 5.74% | 11.78% | 10.35% | 10.78% | 10.40% | 8.81% |
| Educational attainment |  |  |  |  |  |  |
| < high school | 21.49% | 36.26% | 44.81% | 37.50% | 48.41% | 46.61% |
| High school | 19.01% | 30.22% | 24.72% | 29.81% | 22.61% | 22.62% |
| > high school | 59.50% | 33.52% | 30.46% | 32.69% | 28.99% | 30.77% |
| Number of siblings |  |  |  |  |  |  |
| 0 | 12.40% | 21.15% | 27.95% | 35.24% | 27.56% | 31.88% |
| 1-3 | 58.68% | 50.00% | 54.37% | 46.67% | 51.70% | 48.03% |
| 4+ | 28.93% | 28.85% | 17.69% | 18.10% | 20.74% | 20.09% |
| Number of children |  |  |  |  |  |  |
| 0 | 9.84% | 6.54% | 8.26% | 8.57% | 9.63% | 11.54% |
| 1-3 | 59.84% | 53.68% | 53.48% | 53.33% | 55.52% | 48.29% |
| 4+ | 30.33% | 39.78% | 38.26% | 38.10% | 34.84% | 40.17% |
| Medicare drug coverage | 66.94% | 68.97% | 71.23% | 63.54% | 67.31% | 69.34% |
| Medicaid | 21.49% | 22.56% | 26.92% | 25.25% | 30.42% | 41.86% |
| Tricare | 8.26% | 6.08% | 4.46% | 5.94% | 4.09% | 3.17% |
| Number of Comorbidities |  |  |  |  |  |  |
| 0 | 15.70% | 12.43% | 7.05% | 11.88% | 4.93% | 3.07% |
| 1-3 | 71.90% | 68.78% | 65.42% | 53.47% | 57.97% | 6.87% |
| 4+ | 12.40% | 18.78% | 27.53% | 34.65% | 37.10% | 36.40% |
| Marital status |  |  |  |  |  |  |
| Never married | 4.96% | 3.55% | 4.13% | 4.76% | 5.95% | 6.87% |
| Married/live with a partner | 63.64% | 47.54% | 36.96% | 39.05% | 30.59% | 28.76% |
| Separated, divorced, widowed | 31.40% | 48.91% | 58.91% | 56.19% | 63.46% | 64.38% |
| Vigorous activity | 50.00% | 29.43% | 24.84% | 19.05% | 14.73% | 8.58% |
| Smoking regularly | 44.90% | 51.34% | 47.62% | 48.75% | 37.83% | 34.41% |
| MCI | 63.11% | 57.22% | 42.61% | 20.00% | 35.13% | 14.10% |
| Sample size | 122 | 367 | 460 | 105 | 353 | 234 |

Note: Mean (SD) for continuous variables and % for categorical variables.

Table S7. Group-based assessment-based trajectory model of general health status

| Group | Parameter | Estimate | Standard Error | T for H0:  Parameter=0 | Prob>\|T\| |
| --- | --- | --- | --- | --- | --- |
| 1 | Intercept | 86.83 | 0.85 | 101.64 | 0.00 |
|  | Linear | -0.85 | 0.21 | -3.97 | 0.00 |
| 2 | Intercept | 102.13 | 3.31 | 30.84 | 0.00 |
|  | Linear | -26.72 | 2.27 | -11.77 | 0.00 |
|  | Quadratic | 2.70 | 0.30 | 9.09 | 0.00 |
| 3 | Intercept | 13.34 | 3.88 | 3.44 | 0.00 |
|  | Linear | 23.83 | 2.08 | 11.44 | 0.00 |
|  | Quadratic | -2.53 | 0.25 | -10.29 | 0.00 |
| 4 | Intercept | 22.17 | 1.39 | 15.94 | 0.00 |
|  | Linear | 0.53 | 0.36 | 1.46 | 0.14 |

Table S8. Baseline sample characteristics by trajectory group of general health status

|  | High start, slight decrease | High start, convex | Low start, concave | Low start, slight increase |
| --- | --- | --- | --- | --- |
| Female | 61.94% | 58.02% | 59.92% | 57.91% |
| Age | 81.61 (7.66) | 81.64 (7.82) | 80.20 (7.16) | 79.86 (7.34) |
| Race/Ethnicity |  |  |  |  |
| White | 65.67% | 54.38% | 46.92% | 42.76% |
| Black | 25.36% | 29.64% | 36.54% | 36.32% |
| Other | 3.65% | 3.09% | 5.77% | 4.37% |
| Hispanic | 5.32% | 12.89% | 10.77% | 16.55% |
| Educational attainment |  |  |  |  |
| < high school | 31.62% | 41.86% | 55.00% | 60.69% |
| High school | 26.70% | 25.32% | 27.31% | 18.62% |
| > high school | 41.68% | 32.82% | 17.69% | 20.69% |
| Number of siblings |  |  |  |  |
| 0 | 26.84% | 29.56% | 25.48% | 22.92% |
| 1-3 | 54.90% | 50.90% | 46.33% | 49.44% |
| 4+ | 18.26% | 19.54% | 28.19% | 27.64% |
| Number of children |  |  |  |  |
| 0 | 10.58% | 8.14% | 10.31% | 8.46% |
| 1-3 | 54.42% | 57.00% | 52.67% | 49.44% |
| 4+ | 35.01% | 34.86% | 37.02% | 42.09% |
| Medicare drug coverage | 68.70% | 66.85% | 70.33% | 72.20% |
| Medicaid | 20.46% | 28.38% | 30.04% | 40.28% |
| Tricare | 5.62% | 3.90% | 3.50% | 3.26% |
| Number of Comorbidities |  |  |  |  |
| 0 | 11.50% | 9.69% | 3.11% | 2.96% |
| 1-3 | 73.01% | 62.30% | 59.14% | 48.06% |
| 4+ | 15.49% | 28.01% | 37.74% | 48.97% |
| Marital status |  |  |  |  |
| Never married | 5.24% | 4.62% | 6.11% | 5.35% |
| Married/live with a partner | 37.99% | 36.67% | 38.55% | 38.31% |
| Separated, divorced, widowed | 56.77% | 58.72% | 55.34% | 56.35% |
| Vigorous activity | 28.93% | 21.74% | 16.03% | 12.03% |
| Smoking regularly | 43.29% | 44.92% | 44.29% | 51.99% |
| MCI | 43.84% | 33.84% | 35.88% | 34.08% |
| Sample size | 917 | 393 | 262 | 449 |

Note: Mean (SD) for continuous variables and % for categorical variables.

Table S9. Comparison of the individuals included in the sample with individuals excluded from the sample because of having fewer than three rounds of observations

|  | *Physical functioning* Individuals excluded from the sample (v.s. individuals included) | *General Health Status* Individuals excluded from the sample (v.s. individuals included) |
| --- | --- | --- |
| Female | 0.01 | -0.09 |
|  | (-0.26, 0.28) | (-0.45, 0.26) |
| Age | 0.11 | 0.27 |
|  | (-0.17, 0.40) | (-0.11, 0.68) |
| Age squared | -0.00 | -0.00 |
|  | (-0.00, 0.00) | (-0.00, 0.00) |
| Race/Ethnicity (Ref. White) | |  |
| Black | -0.02 | -0.02 |
|  | (-0.31, 0.26) | (-0.39, 0.34) |
| Other | -0.71 | -0.98 |
|  | (-1.58, 0.04) | (-2.43, 0.10) |
| Hispanic | -0.24 | -0.61 |
|  | (-0.71, 0.21) | (-1.35, 0.05) |
| Educational attainment (Ref. < High school) | |  |
| High school | -0.32* | -0.02 |
|  | (-0.62, -0.02) | (-0.40, 0.36) |
| > High school | -0.41** | -0.21 |
|  | (-0.70, -0.13) | (-0.58, 0.15) |
| # siblings (Ref. 0) |  |  |
| 1-3 | -0.13 | -0.21 |
|  | (-0.39, 0.14) | (-0.53, 0.11) |
| 4+ | -0.25 | -0.58* |
|  | (-0.60, 0.11) | (-1.09, 0.10) |
| # children (Ref. 0) |  |  |
| 1-3 | -0.21 | 0.21 |
|  | (-0.61, 0.20) | (-0.33, 0.79) |
| 4+ | -0.45* | -0.14 |
|  | (-0.88, -0.02) | (-0.71, 0.46) |
| Medicare drug coverage | -0.31* | -0.38* |
|  | (-0.56, -0.05) | (-0.72, -0.05) |
| Medicaid | 0.24 | 0.00 |
|  | (-0.06, 0.54) | (-0.38, 0.40) |
| Tricare | 0.25 | -0.29 |
|  | (-0.06, 0.54) | (-0.99, 0.51) |
| Comorbidity (Ref. 0) |  |  |
| 1-3 | 0.38 | 0.49 |
|  | (-0.09, 0.88) | (-0.16, 1.25) |
| 4+ | 0.53* | 0.80* |
|  | (0.04, 1.06) | (0.12, 1.57) |
| Marital status (Ref. Never married) |  |  |
| Married/live with a partner | -0.49 | -0.08 |
|  | (-1.07, 0.11) | (-0.90, 0.82) |
| Separated, divorced, widowed | -0.47 | -0.09 |
|  | (-1.04, 0.10) | (-0.87, 0.79) |
| Smoking regularly | -0.21 | -0.17 |
|  | (-0.45, 0.03) | (-0.48, 0.13) |
| MCI | -0.78*** | -1.09*** |
|  | (-1.04, -0.53) | (-1.47, -0.72) |

Note: * p<0.05; ** p<0.01; *** p<0.001. N = 1,634. Because of missing control variables, two models have the same sample sizes.
